# The *Arabidopsis* Thylakoid Chloride Channel AtCLCe Functions in Chloride Homeostasis and Regulation of Photosynthetic Electron Transport

**DOI:** 10.3389/fpls.2016.00115

**Published:** 2016-02-09

**Authors:** Andrei Herdean, Hugues Nziengui, Ottó Zsiros, Katalin Solymosi, Győző Garab, Björn Lundin, Cornelia Spetea

**Affiliations:** ^1^Department of Biological and Environmental Sciences, University of GothenburgGothenburg, Sweden; ^2^Biological Research Center, Hungarian Academy of SciencesSzeged, Hungary; ^3^Department of Plant Anatomy, Eötvös Loránd UniversityBudapest, Hungary

**Keywords:** *Arabidopsis thaliana*, CLC channel, chlorophyll fluorescence, electron microscopy, photosynthetic electron transport, proton motive force, state transition, thylakoid membrane

## Abstract

Chloride ions can be translocated across cell membranes through Cl^−^ channels or Cl^−^/H^+^ exchangers. The thylakoid-located member of the Cl^−^ channel CLC family in *Arabidopsis thaliana* (AtCLCe) was hypothesized to play a role in photosynthetic regulation based on the initial photosynthetic characterization of *clce* mutant lines. The reduced nitrate content of *Arabidopsis clce* mutants suggested a role in regulation of plant nitrate homeostasis. In this study, we aimed to further investigate the role of AtCLCe in the regulation of ion homeostasis and photosynthetic processes in the thylakoid membrane. We report that the size and composition of proton motive force were mildly altered in two independent *Arabidopsis clce* mutant lines. Most pronounced effects in the *clce* mutants were observed on the photosynthetic electron transport of dark-adapted plants, based on the altered shape and associated parameters of the polyphasic *OJIP* kinetics of chlorophyll *a* fluorescence induction. Other alterations were found in the kinetics of state transition and in the macro-organization of photosystem II supercomplexes, as indicated by circular dichroism measurements. Pre-treatment with KCl but not with KNO_3_ restored the wild-type photosynthetic phenotype. Analyses by transmission electron microscopy revealed a bow-like arrangement of the thylakoid network and a large thylakoid-free stromal region in chloroplast sections from the dark-adapted *clce* plants. Based on these data, we propose that AtCLCe functions in Cl^−^ homeostasis after transition from light to dark, which affects chloroplast ultrastructure and regulation of photosynthetic electron transport.

## Introduction

Photosynthesis is essential for life on Earth. A key element of photosynthesis is conversion of sunlight energy into organic carbon via the generation of a membrane electrochemical potential gradient for protons (H^+^), also known as the proton motive force (PMF). To generate PMF, pigments (chlorophylls and carotenoids) bound to proteins in light harvesting complexes (LHCs) absorb photons and transfer their excitation energy to the reaction centers of photosystems (PS). Here the excitation is converted into charge separation, which drives electron transport from PSII to PSI via the cytochrome b_6_*f* (Cyt b_6_*f*) complex. The net result of this process is the oxidation of water molecules (oxygen evolution) and the reduction of NADP^+^, which is associated with translocation of H^+^ into the thylakoid lumen. A light-driven cyclic electron transport around PSI and Cyt b_6_*f* is also operative, which does not evolve oxygen, nor induce NADP^+^ reduction but only contributes to H^+^ translocation. It is the water oxidation and photosynthetic electron transport-coupled H^+^ translocation into the lumen that generates PMF, which is composed of transmembrane H^+^ concentration (ΔpH) and electrical potential (ΔΨ) gradients. Both PMF components can drive ATP synthesis, whereas it is thought that only the ΔpH component can activate the photoprotective PsbS- and xanthophyll cycle-dependent components of non-photochemical quenching (*NPQ*) while down-regulating the electron transport during the step of plastoquinol oxidation at Cyt b_6_*f* complex (Kramer et al., 2003). The role of ΔΨ in regulation of photosynthesis is less clear, but there is recent evidence in *Arabidopsis thaliana* (hereafter referred to as *Arabidopsis*) that ion channels, such as the thylakoid K^+^ channel TPK3, partially dissipate ΔΨ to allow more H^+^ to enter the lumen and thus a significant ΔpH to be formed, balancing photoprotection and photochemical efficiency (Carraretto et al., 2013).

Potassium, chloride, magnesium, and calcium are the major ions in thylakoids, and changes in their homeostasis are expected to impact membrane architecture, protein conformation, and electron transport rates (Anderson et al., 2012; Pribil et al., 2014; Finazzi et al., 2015; Pottosin and Shabala, 2015). Evidence for fluxes of these ions across the spinach thylakoid membrane accompanying the inward movement of H^+^ during electron transport reactions was provided already by Hind et al. (1974). The proteins involved in K^+^ fluxes have been recently characterized in *Arabidopsis* (Carraretto et al., 2013; Armbruster et al., 2014; Kunz et al., 2014). Voltage-dependent chloride channel activities in the thylakoid membrane have been reported in *Peperomia metallica* (Schönknecht et al., 1988) and in the alga *Nitellopsis obtusa* (Pottosin and Schönknecht, 1995), but thus far the proteins responsible for those activities have not been identified.

In plants, three gene families for Cl^−^ transport have been described thus far, namely slow-anion channels (SLAC), aluminum-activated malate transporters (ALMT), and Cl^−^ channels (CLCs; Barbier-Brygoo et al., 2011). The *Arabidopsis* genome codes for seven CLC members (AtCLCa to AtCLCg), localized in various intracellular membrane compartments, and thought to be either Cl^−^ channels or Cl^−^/H^+^ exchangers. Nevertheless, several of them have been also reviewed as nitrate transporters (Krapp et al., 2014). More specifically, AtCLCa and AtCLCb are tonoplast-located 2${\text{NO}}_{3}^{-}$/1H^+^ antiporters (De Angeli et al., 2006; von der Fecht-Bartenbach et al., 2010). AtCLCc and AtCLCg are also located in the tonoplast, AtCLCd, and AtCLCf in the Golgi apparatus, and AtCLCe in the thylakoid membrane (Teardo et al., 2005; Marmagne et al., 2007; von der Fecht-Bartenbach et al., 2007; Lv et al., 2009). The selectivity and mechanism of anion transport for these five AtCLCs are unknown (Barbier-Brygoo et al., 2011).

Various physiological functions have been proposed for AtCLCs, based on the phenotypic characterization of corresponding *Arabidopsis* knockout mutants. AtCLCa, AtCLCb, and AtCLCe are required to maintain normal cellular ${\text{NO}}_{3}^{-}$ levels (De Angeli et al., 2007; von der Fecht-Bartenbach et al., 2010), and in addition AtCLCe may regulate the photosynthetic activity of thylakoids (Marmagne et al., 2007). AtCLCc participates in both ${\text{NO}}_{3}^{-}$ and Cl^−^ homeostasis, and regulates stomatal movement and salt tolerance (Jossier et al., 2010). AtCLCg is also involved in salt tolerance by altering Cl^−^ homeostasis in mesophyll cells (Nguyen et al., 2015). AtCLCd has been proposed to regulate lumenal pH in the trans-Golgi network (von der Fecht-Bartenbach et al., 2007), and to act as a negative regulator of plant innate immunity (Guo et al., 2014). The physiological function of AtCLCf is still unknown.

In this study, we addressed the question about the physiological role of AtCLCe in the thylakoid membrane. We show that AtCLCe loss-of-function mutation modifies the arrangement of thylakoid network in chloroplasts and alters photosynthetic electron transport following transfer from light to dark.

## Materials and methods

### Plant growth conditions

*A. thaliana* cv. Columbia (Col-0) plants and two *clce* mutants in the same background were grown in soil for 7–8 weeks in a growth chamber (CLF PlantMaster, Plant Climatics, Wertingen, Germany) using 8-h-light (120 μmol photons m^−2^ s^−1^)/16-h-dark cycles at 22°C and 70% relative humidity. The SALK_010237 *(clce-2)* and SALK_21945C (*clce-3)* mutants were obtained from the Arabidopsis Biological Resource Center (ABRC, https://www.arabidopsis.org/abrc/). The *clce-2* line was previously characterized by Marmagne et al. (2007).

### RNA isolation and RT-PCR

Total RNA was isolated from rosette leaves of 7-8-week old plants using TriZol reagent (Invitrogen), treated with RNase-free DNAse (Thermo Scientific) to prevent DNA contamination and then purified using HiBind RNA mini columns (Omega Bio-Tek) according to the manufacturer's instructions. cDNA was synthesized using 1 μg of total RNA through iScript cDNA synthesis Kit (Bio-Rad). Finally, 2 μL of reverse transcription reaction were used as template to amplify *AtCLCe* and β*-ATPase* cDNA fragments using Dream Taq DNA Polymerase Kit (Thermo Scientific). The following primers were used for the *AtCLCe* (*At4g35440*): *forward* TCCAAGTGTTGAAATTGGAGC and *reverse* AGGTGTAACAGTCCATGGCAC, and for mitochondrial ATP synthase β-subunit *(At5g08680)* selected as reference gene: *forward* GATCATGACATCTCTCGAGG and *reverse* TGGTAAGGAGCAAGGAGATC.

### Determination of leaf chlorophyll (Chl) content

Chl content was determined from leaf discs after extraction in 96% (*v/v*) ethanol at 65°C for 10 min followed by spectrophotometry (Lichtenthaler and Wellburn, 1983). The Chl content was expressed per leaf area and per fresh weight.

### Electrochromic band shift (ECS) measurements

ECS measurements were carried out using a Pulse Amplitude Modulated Chl fluorometer (Dual PAM-100, Walz, Effeltrich, Germany) equipped with a P515/535 module (Schreiber and Klughammer, 2008). Leaves of 30 min dark-adapted plants were illuminated with actinic red light at 100 or 650 μmol photons m^−2^ s^−1^ for 2, 5, or 10 min. After each illumination period, the light was switched off and the dark interval relaxation kinetics (DIRK) of the ECS signal were recorded for 60 s according to Cruz et al. (2001) to estimate PMF size (ECS_t_) and relative contribution of ΔpH and ΔΨ to PMF. Before each PMF measurement, a saturating single turnover 5-μs flash of 200,000 μmol photons m^−2^ s^−1^ was applied to determine ECS_ST_, which was used to normalize ECS_t_.

For determination of H^+^ conductivity of the thylakoid membrane mainly through ATP synthase (g_H_^+^), the leaves were exposed to light at 650 μmol photons m^−2^ s^−1^ for 10 min. At specific time points, the light was switched off to record the ECS signal decay during 600-ms dark intervals. The g_H_^+^ parameter was calculated as 1/time constant for decay derived from single exponential fittings of the ECS decay during the first 100 ms (Cruz et al., 2005). The steady state proton flux (ν_H_^+^) was calculated as ${\text{g}}_{\text{H}}^{}$ ^*^ ECS_t_/ECS_ST_ (Cruz et al., 2005).

### Kinetics of Chl *a* fluorescence induction

Fast Chl *a* fluorescence induction (*OJIP*) kinetics were recorded using a Plant Efficiency Analyser (Handy-PEA, Hansatech, King's Lynn, Norfolk, UK) by applying saturating red actinic light (635 nm, 3500 μmol photons m^−2^ s^−1^, 1 s) on plants during dark adaptation intervals of 1–15 min. Where indicated, recorded data points were double normalized to minimum (*F*_**0**_) and maximum (*F*_*m*_) fluorescence. The time to reach *F*_*m*_ (t_Fm_ in ms), the maximum quantum yield of PSII (*F*_*v*_*/F*_*m*_), the performance index (*PI*), variable fluorescence yield at *J* step (*V*_*J*_), variable fluorescence yield at *I* step (*V*_*I*_*)*, and the turnover number of Q_A_ (*N*) were calculated using Hansatech PEA Plus v1.10 software according to Strasser et al. (2004). Where indicated, detached leaves were incubated in 150 mM KCl or KNO_3_ for 30 min in growth light (120 μmol photons m^−2^ s^−1^) followed by adaptation in darkness for 15 min before the *OJIP* measurements.

Slow Chl *a* fluorescence induction and recovery kinetics were recorded using the Dual PAM-100 instrument (Walz) on 30-min dark-adapted plants using red actinic light of 100 or 650 μmol photons m^−2^ s^−1^ for 10 min followed by 5 min in darkness. The non-photochemical quenching (*NPQ*) and quantum yield of PSII [Φ (*II*)] were calculated using the following equations: Φ (*II*) = $(F_{m}{}^{\prime }-F)/F_{m}{}^{\prime }$, where $F_{m}{}^{\prime }$ is defined as the fluorescence value at the plateau level reached during application of a saturating pulse, and *F* is defined as the fluorescence level during illumination averaged for 0.2 s before applying the saturating pulse; $NPQ=(F_{m}-F_{m}{}^{\prime })/F_{m}{}^{\prime }$, where *F*_*m*_ is the fluorescence value at the plateau level reached during application of a saturating pulse on the 30 min dark-adapted leaf before the onset of illumination.

### P700 oxidation-reduction kinetics

To monitor the oxidation-reduction kinetics of PSI, absorbance changes at 830 nm (reflecting the redox state of P700, i.e., PSI primary donor) were recorded using the Dual PAM-100 (Walz) instrument. The 830-nm transmittance was subtracted from the simultaneous recording at 875 nm, and calibrated according to the Dual PAM-100 built-in routine, and finally displayed as P700 ΔI/I^*^10^3^. Before the measurement, plants were adapted to light (120 μmol photons m^−2^ s^−1^, 1 h). Oxidation-reduction kinetics were recorded by applying saturating red actinic light pulses (635 nm, 20,000 μmol photons m^−2^ s^−1^, 200 ms) together with far red (FR) light (730 nm) during the illumination period and after 1, 2, 3, 4, and 5 min of dark adaptation. Half of each dark-adaptation interval (30 s) was done in the presence of FR light to fully oxidize P700 before applying the saturation pulse.

### State transition (ST) kinetics

ST measurements were carried out according to Lunde et al. (2000) on 30 min dark-adapted plants using the Dual PAM-100 (Walz) instrument. For determination of *F*_*m*_ values in the dark and in either state 1 or state 2, a saturating pulse of red actinic light (5000 μmol photons m^−2^ s^−1^, 800 ms) was applied. To induce state 2, leaves were illuminated for 15 min with red actinic light (100 μmol photons m^−2^ s^−1^; “state 2 light”). For transition to state 1, leaves were exposed for 15 min to red light supplemented with FR light (“state 1 light”). The *qT* parameter was calculated as *(F*_*m*2_ − *F*_*m*3_*)/F*_*m*2_, where *F*_*m*2_ is the fluorescence value at the plateau level reached during application of the saturating pulse after 15 min illumination with “state 1 light” and *F*_*m*3_ after 15 min illumination with “state 2 light.” The *qS* parameter was calculated as *[*(*F*${}_{I^{\prime }}$*-F*_*I*_*)-(F*${}_{II^{\prime }}$*-F*_*II*_*)]/(F*${}_{I^{\prime }}$*-F*_*I*_*)* according to Damkjaer et al. (2009), where *F*_*I*_ and *F*_*II*_ are the steady-state fluorescence levels in the presence of FR light in state 1 and state 2, respectively, and *F*${}_{I^{\prime }}$ and *F*${}_{II^{\prime }}$ are the steady-state fluorescence levels in the absence of FR light in state 1 and state 2, respectively.

### Circular dichroism (CD) spectroscopy

CD measurements were carried out on a J-815 spectropolarimeter (JASCO, Tokyo, Japan). Detached, water-infiltrated leaves were placed between two glass slides in an optical cell. Spectra were recorded at room temperature between 400 and 800 nm at a scan speed of 100 nm min^−1^, band-pass of 3 nm and step size of 1 nm. For each sample, 3–4 scans were averaged. Spectra were normalized to the absorption of the red-most peak of the spectra recorded at the same time as the CD spectra and were corrected for baseline distortions. Measurements were repeated on three different leaves for each genotype. Amplitudes of psi-type CD bands, at around (+)505, (−)675, and (+)690 nm, were determined using the reference wavelengths of 550, 600, and 750 nm, respectively.

### Transmission electron microscopy (TEM)

Sections (~2 × 2 mm) cut from the central parts of leaf blades of 7-week-old plants were fixed in 2.5% (*v/v*) glutaraldehyde for 4 days, then post-fixed in 1% OsO_4_ (*w/v*) for 2 h. Fixatives were buffered with 70 mM Na_2_HPO_4_-KH_2_PO_4_ (pH 7.2). After fixation, samples were rinsed in the same buffer. After dehydration in an alcohol series, samples were embedded in Durcupan ACM resin (Fluka, Buchs, Switzerland). Ultrathin sections (thickness 70 nm) were cut with a Reichert Jung Ultracut E microtome (Reichert-Jung AG, Vienna, Austria), mounted on copper grids and contrasted with 5% uranyl acetate and Reynolds' lead citrate solution. The sections were visualized with a Hitachi 7100 TEM microscope at 75 kV accelerating voltage.

ImageJ software was used to measure granum diameter (at the middle of perpendicular granum sections) on the micrographs. Calculations were done on 210-350 randomly chosen grana originating from 35 different chloroplasts taken randomly from 35 different mesophyll cells per treatment.

### Statistical analyses

Each mean is given ± SEM for at least 5 plants. The data sets were compared between genotypes by one-way ANOVA using OriginPro 8 for Windows. Significant differences were considered at *P* < 0.05.

## Results

### Phenotype of *clce* mutants

**Figure 1:**
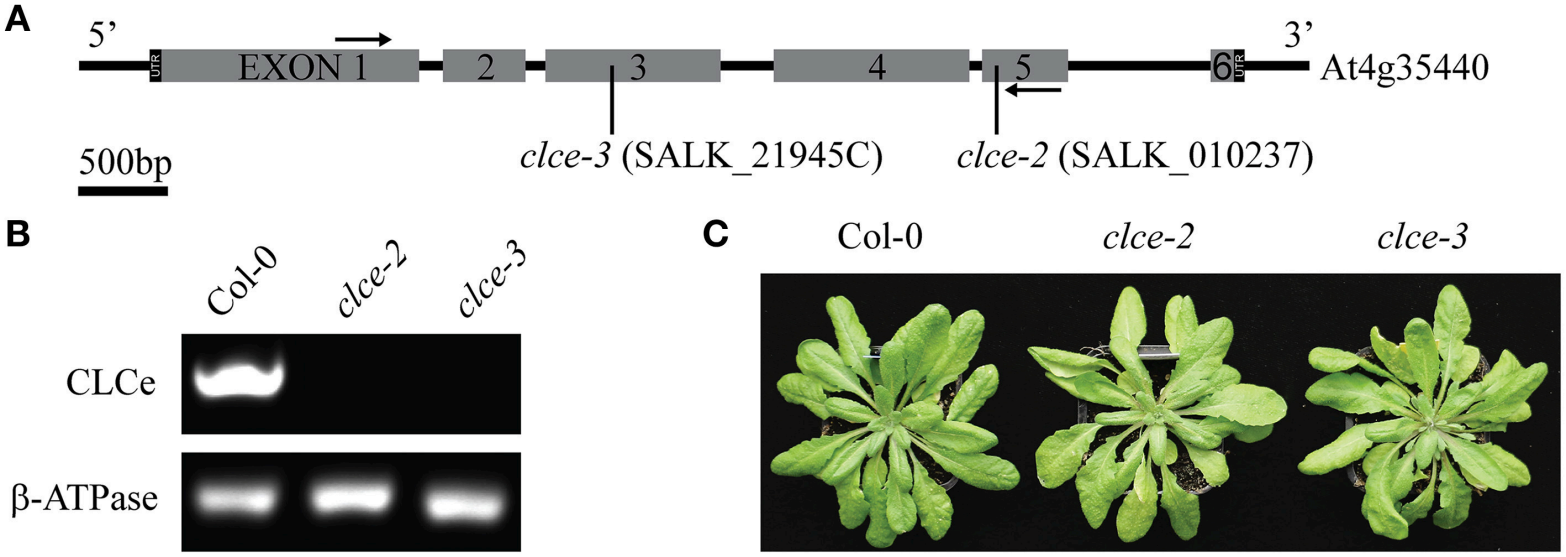
**Genotyping of ***clce*** mutants used in this study**. **(A)** *AtCLCe* gene structure with indicated location of T-DNA insert in each of the two *clce* lines. Black arrows indicate location of primers used for RT-PCR. **(B)** RT-PCR of total RNA from wild-type (Col-0) and *clce* leaves with primers specific for the *AtCLCe* gene and primers for a reference gene as the positive control. **(C)** Representative photos of 7-week-old plants.

To investigate the role of AtCLCe in the thylakoid membrane, we have characterized the phenotype of two *Arabidopsis* T-DNA insertion lines in Col-0 background. The *clce-2* mutant was initially characterized by Marmagne et al. (2007). We have identified an additional T-DNA insertion line (*clce-3*) in the same background (Figure 1A). Both genotypes lacked the *AtCLCe* transcript, as revealed by RT-PCR analyses (Figure 1B). Neither line displayed obvious growth differences from wildtype (Figure 1C) and no statistically significant differences in Chl content, Chl *a/b* ratio or specific weight of leaves were obtained (Table 1).

**Table 1:** **Chlorophyll (Chl) content and photosynthetic performance of dark-adapted plants**.

**Genotype**	**Col-0**	***clce-2***	***clce-3***
mg Chl cm^−2^	0.024±0.001	0.022±0.001	0.022±0.001
mg Chl g^−1^ fresh weight	1.335±0.075	1.232±0.073	1.248±0.043
mg fresh weight cm^−2^	18.100±0.693	17.670±0.403	17.490±0.429
Chl *a/b*	4.077±0.042	4.065±0.051	4.027±0.029
*F_0_*	486±5	500±10	518±6
*F_*m*_*	2624±21	2442±2^*^	2504±18^*^
*F_*v*_/F_*m*_*	0.814±0.001	0.795±0.001^*^	0.795±0.001^*^
*PI*	2.503±0.044	2.037±0.042^*^	2.105±0.036^*^
*V_*J*_*	0.368±0.002	0.341±0.002^*^	0.337±0.001^*^
*V_*I*_*	0.750±0.007	0.598±0.003^*^	0.610±0.007^*^
*t_*Fm*_* (ms)	338±43	600±49^*^	580±43^*^
*N*	59.493±1.742	127.314±1.633^*^	122.545±3.991^*^

*The wild-type (Col-0) plants and clce mutants were grown for 7 weeks using a 16 h dark/8 h light (120 μmol photons m^−2^ s^−1^) photoperiod. Leaf Chl content and a/b ratio were determined from leaf discs of 16-h dark-adapted plants following extraction in ethanol and spectrophotometry. Fast kinetics of Chl a fluorescence were recorded on 15 min dark-adapted plants with Handy-PEA. The following parameters were calculated with the JIP test: the minimum chlorophyll fluorescence (F_0_), maximum fluorescence (F_m_), the maximum quantum yield (F_v_/F_m_), the performance index (PI), the relative variable fluorescence yield at 2 ms (V_J_) and at 30 ms (V_I_), the time to reach F_m_ (t_Fm_), and the turnover number of Q_A_(N). The data are means ± SEM (n = 5 plants). Asterisks indicate significant difference in the studied parameters between Col-0 and the clce mutants (ANOVA, P < 0.05)*.

Next we compared the photosynthetic performance of dark-adapted plants based on Chl *a* fluorescence induction parameters. The *F*_*v*_*/F*_*m*_ parameter used as an indicator of the maximum quantum yield of PSII was found slightly but significantly lower in 15 min dark-adapted *clce* mutants than in wildtype due to a significantly lower *F*_*m*_ (Table 1). Another Chl fluorescence parameter used as indicator of the overall plant vitality, is the performance index (*PI*) of dark-adapted plants (Strasser et al., 2000). *PI* was found significantly reduced in the *clce* mutants, suggesting that they experience stress during dark-adaptation. These data indicate that AtCLCe loss-of-function mutation lowers photosynthetic performance in dark-adapted plants.

### Proton motive force and proton flux

**Figure 2:**
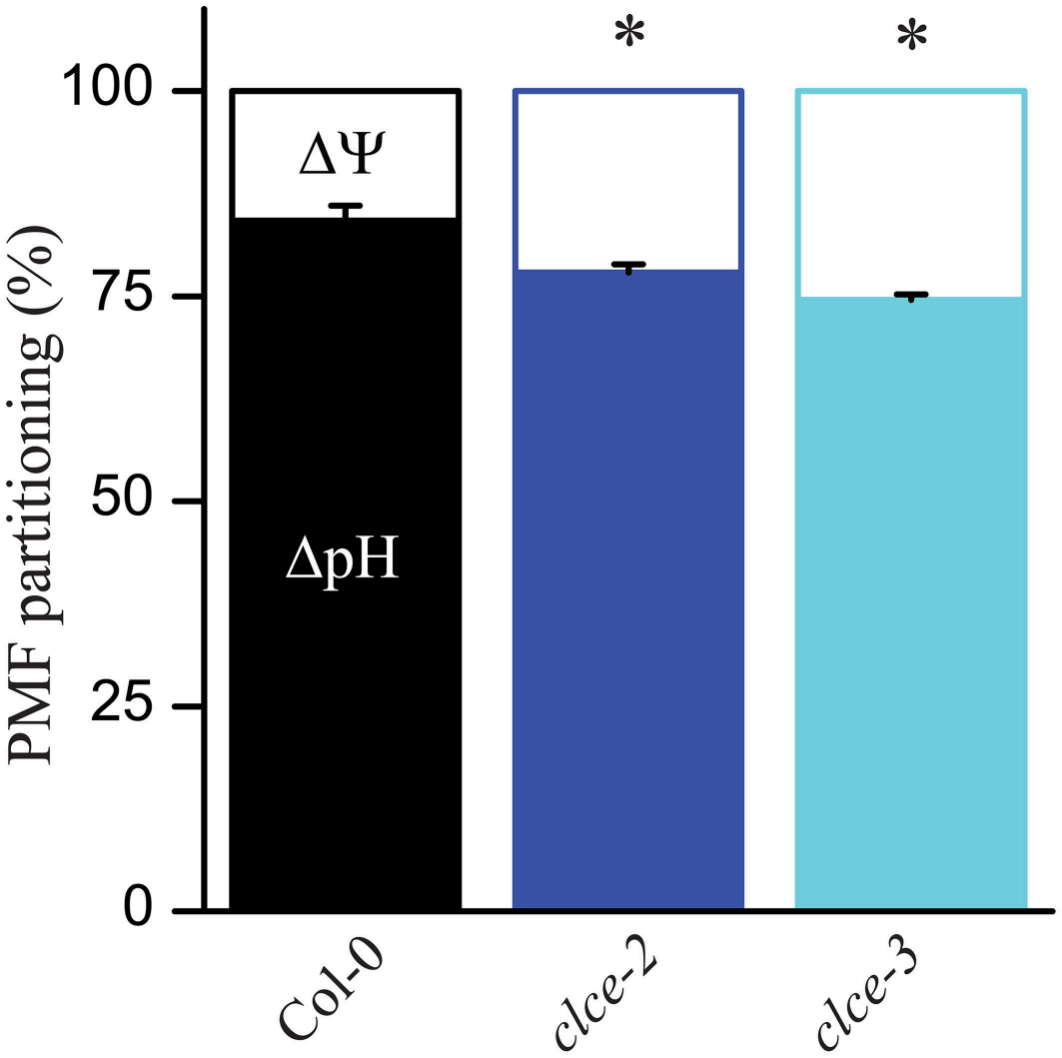
**Composition of the proton motive force (PMF)**. Wild-type and mutant plants were dark-adapted for 30 min and then illuminated for 10 min with 650 μmol photons m^−2^ s^−1^. Following illumination, kinetics of dark relaxation of the electrochromic shift (ECS) signal were recorded and deconvoluted to determine membrane pH gradient (ΔpH) and membrane potential (ΔΨ) components. The plotted data are means ± SEM (*n* = 8–9 plants). Asterisks indicate statistically significant differences according to ANOVA (*P* < 0.05).

To determine if PMF partitioning into ΔpH and ΔΨ has been altered in the *clce* mutants as compared to wildtype, leaves were illuminated for 10 min at either 100 or 650 μmol photons m^−2^ s^−1^ and the DIRK of ECS were recorded according to Cruz et al. (2001). In the mutants, a mild but significant increase in ΔΨ was observed at both light intensities, and consequently a decrease in ΔpH occurred (Figure 2 and Supplementary Figure 1A). This suggests a minor alteration by the AtCLCe loss-of-function in the ion distribution across the thylakoid membrane during illumination. It is of note that differences in PMF partitioning were also observed at 5 min but not after shorter illumination at 650 μmol photons m^−2^ s^−1^ (Supplementary Figure 1B).

**Figure 3:**
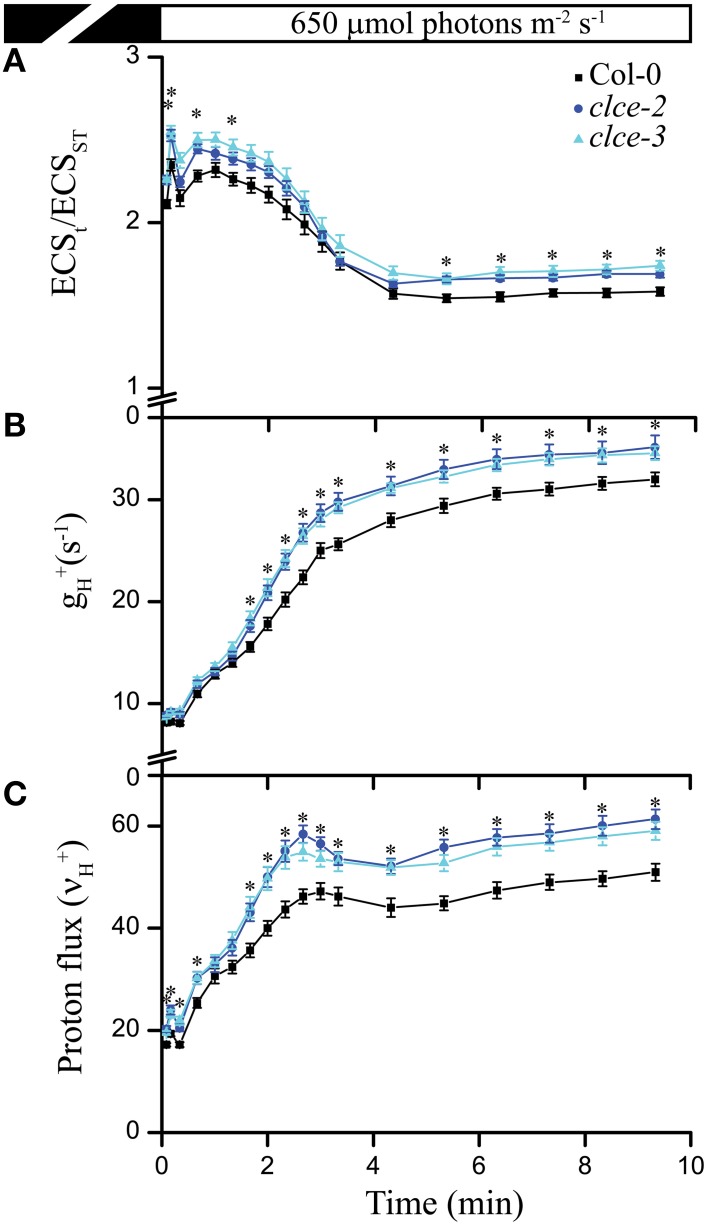
**Proton motive force, H^**+**^ conductivity through ATP synthase, and H^**+**^ flux**. Electrochromic shift measurements (ECS) were performed on 30 min dark-adapted wild-type and mutant plants. **(A)** Total proton motive force (ECS_t_ normalized to ECS_ST_) induction kinetics during illumination at the given light intensity. **(B)** ATP synthase conductivity (g_H_^+^), and **(C)** proton flux (ν_H_^+^) were calculated from ECS decay kinetics as described in Section Materials and Methods. The plotted data are means ± SEM (*n* = 8–9 plants). Asterisks indicate statistically significant differences according to ANOVA (*P* < 0.05).

Next we investigated the effect of AtCLCe loss-of-function mutation on ATP synthase activity by measuring the thylakoid membrane total PMF (ECS_t_), conductivity to H^+^ (g_H_^+^), and the H^+^ flux through ATP synthase (ν_H_^+^) during exposure for 10 min to 650 μmol photons m^−2^ s^−1^. Both mutant lines displayed a mild but significant increase in all three parameters throughout the illumination time (Figures 3A–C), suggesting enhanced thylakoid membrane H^+^ conductivity. Since the H^+^ flux is known to linearly correlate with the rate of linear electron flow (Takizawa et al., 2008), the absence of AtCLCe appeared to have a beneficial effect on electron transfer rate in thylakoids during illumination.

### Photoprotection and PSII efficiency

**Figure 4:**
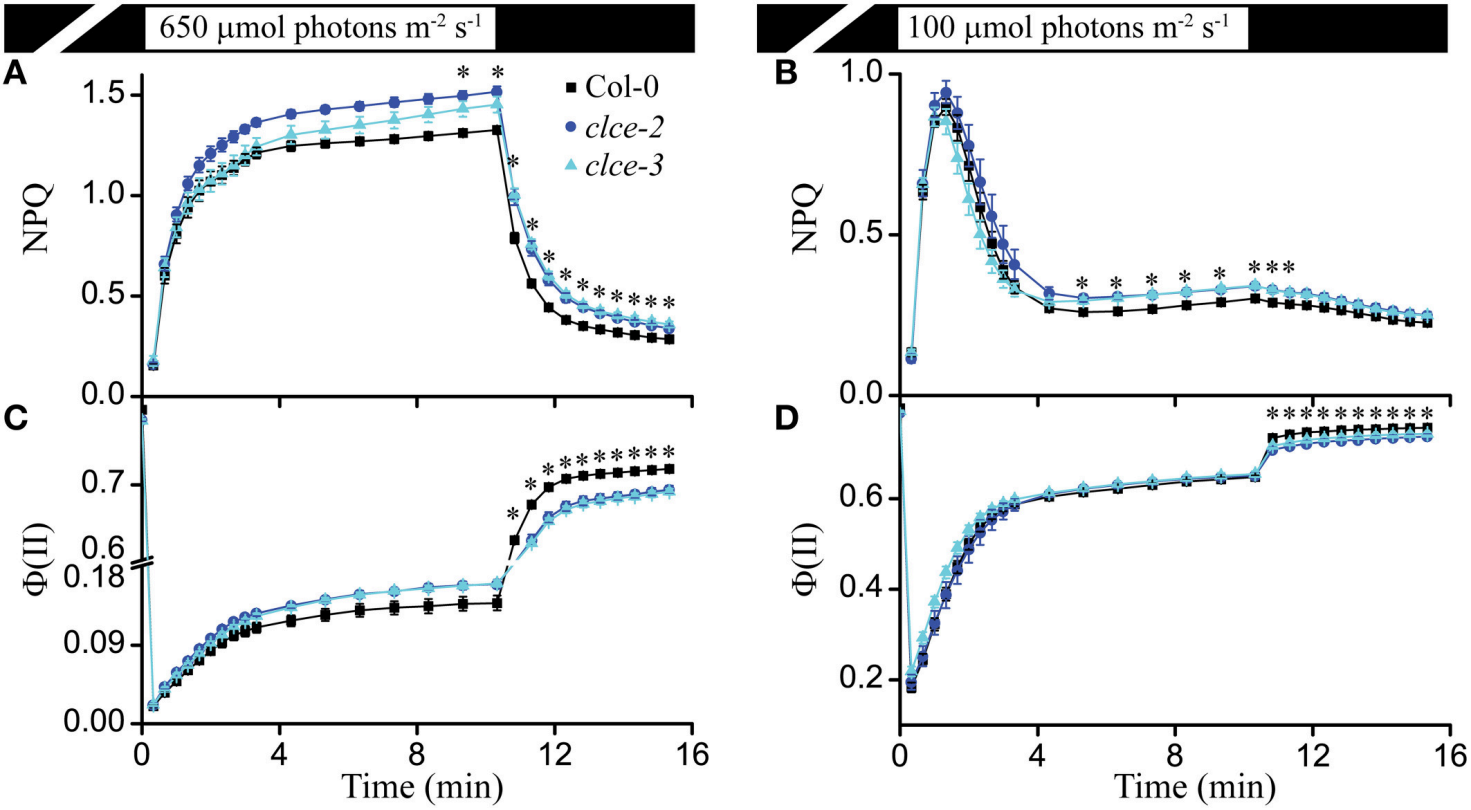
**Non-photochemical quenching (***NPQ***) and PSII efficiency [Φ***(II***)]**. *NPQ* induction kinetics of wild-type and *clce* plants were recorded during illumination for 10 min at 650 μmol photons m^−2^ s^−1^ **(A)** or 100 μmol photons m^−2^ s^−1^ **(B)** followed by 5 min relaxation in darkness. Φ (*II*) from the same set of measurements is plotted in **(C,D)**. The plotted data are means ± SEM (*n* = 9 plants). Asterisks indicate statistically significant differences according to ANOVA (*P* < 0.05).

The ΔpH component of PMF regulates PSII efficiency [Φ (*II*)] and *NPQ* induction with respect to its fast energy-dependent and slow zeaxanthin-dependent quenching components (Nilkens et al., 2010; Ruban et al., 2012). Based on the lower contribution of ΔpH to PMF (Figure 2), we expected a higher Φ (*II*) and lower *NPQ* in the mutants as compared to wildtype. Instead, we observed a significantly higher steady-state *NPQ* level in *clce* after about 10 min of illumination at 650 and 100 μmol photons m^−2^ s^−1^ (Figures 4A,B). The Φ (*II*) parameter did not significantly differ between wildtype and mutants during the same set of measurements (Figures 4C,D). *NPQ* relaxation during the subsequent dark phase after either 650 or 100 μmol photons m^−2^ s^−1^ was significantly slower, whereas Φ (*II*) was consistently lower without reaching wild-type levels (Figures 4A–D). The observed effects of AtCLCe loss-of-function mutation on *NPQ* during illumination may have other cause than the altered relative contribution of ΔpH to the PMF. The lower Φ (*II*) after transition from light to dark is in line with the lower *F*_*v*_*/F*_*m*_ and *PI* of dark-adapted plants (Table 1).

### Fast Chl fluorescence induction and P700 oxidation-reduction kinetics

**Figure 5:**
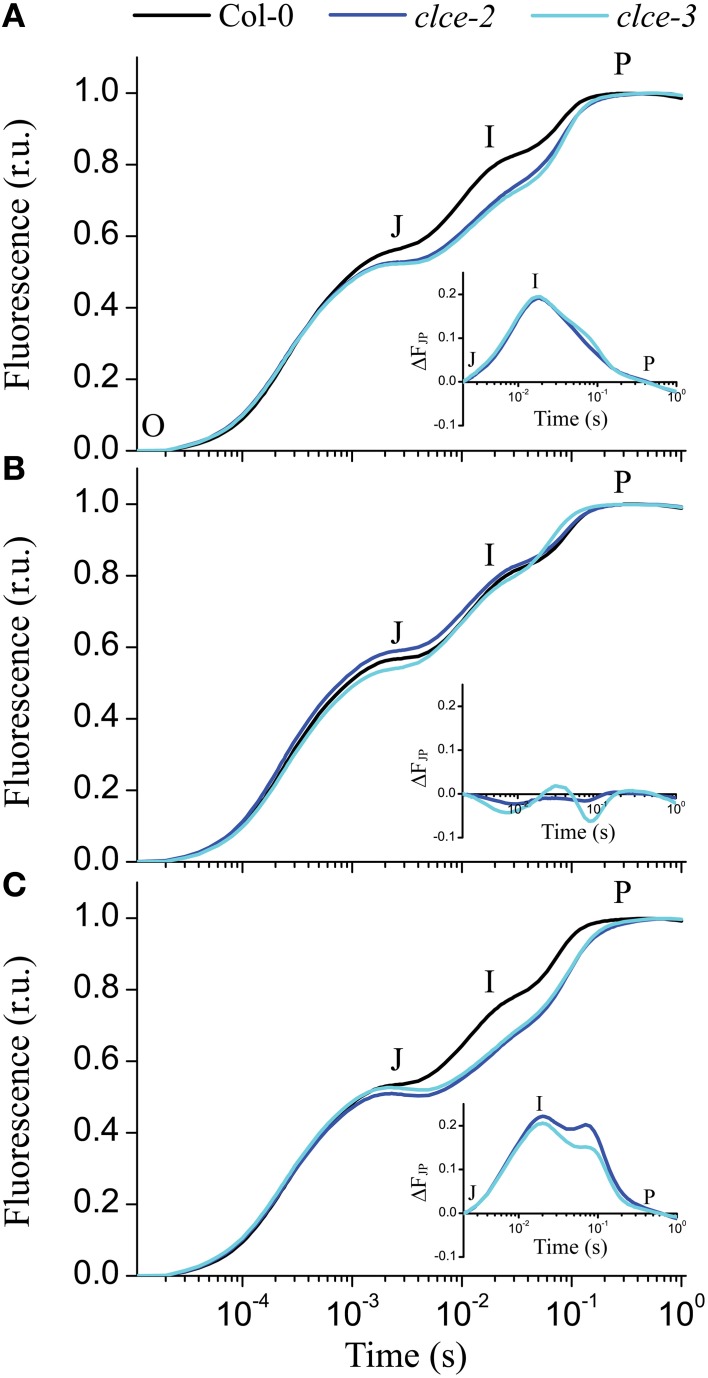
**Fast chlorophyll ***a*** fluorescence ***OJIP*** transients of untreated and salt-treated leaves**. Detached wild-type and mutant leaves that have been incubated in the light for 30 min in water **(A)**, 150 mM KCl **(B)**, or 150 mM KNO_3_ **(C)** were removed from the solution and were dark adapted for 15 min before *OJIP* transients were recorded. The transients were double normalized to *F*_0_ and *F*_*m*_. The time points for the calculation of the *OJIP* parameters are marked: *O*—the fluorescence intensity at 50 μs, *J*—at 2 ms, *I*—at 30 ms, and *P*—the maximum fluorescence intensity. Insets in each plot show the curve difference of double normalized data at *J* and *P* (ΔF_JP_) between wildtype and each of the *clce* mutants. The plotted data are means ± SEM (*n* = 5 plants).

The fast kinetics of Chl *a* fluorescence induction (*OJIP*) display a polyphasic shape and provide information about the electron transport reactions in the thylakoid membrane (Strasser et al., 2000). The shape of the recorded *OJIP* kinetics was found altered in 15-min dark-adapted *clce* mutants (Figure 5A), confirming previous observations (Marmagne et al., 2007). The mutants displayed lower *F*_*v*_*/F*_*m*_ values due to lower fluorescence level at the *P* step (*F*_*m*_, Table 1). The differences in fluorescence levels appeared already at the *J* step and become greater at the *I* step (Figure 5A), corresponding to *V*_*J*_ and *V*_*I*_ parameters (Table 1). The curves were double normalized to *J* and *P*, and the curve difference (ΔF_JP_) between wildtype and *clce* mutants was plotted (Figure 5A inset). The resulting peak corresponds to the *I* step, which has been associated with the redox state of the donor and acceptor side of PSI (Schansker et al., 2005). Marmagne et al. (2007) proposed that the alteration of the *I* peak is due to the disappearance of a rate limiting step in electron transport between the acceptor side of PSII and of PSI. We also found that the time to reach *F*_*m*_ (*t*_*Fm*_) was twice longer in the *clce* mutants than in wildtype (Table 1). The *N* parameter for the number of turnovers of PSII primary electron acceptor (Q_A_), i.e., number of times Q_A_ has been reduced in the interval between 0 and *t*_*Fm*_, was twice as high in the mutants (Table 1). This is in line with the lower fluorescence at the *J* step, which is also related to Q_A_ reduction (Strasser et al., 2000).

Interestingly, pre-treatment of leaves with 150 mM KCl in the light followed by 15 min dark-adaptation resulted in similar *OJIP* kinetics in wildtype and mutants (Figure 5B and inset). Moreover, pre-treatment with KNO_3_ did not rescue the phenotype observed in the *clce* mutants (Figure 5C). Instead, the treatment induced a shoulder after the *I* peak (Figure 5C inset) of unclear origin. The restoration of *OJIP* wild-type shape in the *clce* mutants by KCl treatment suggests that the untreated samples had altered kinetics due to a disturbed Cl^−^ distribution across thylakoids in dark-adapted plants.

**Figure 6:**
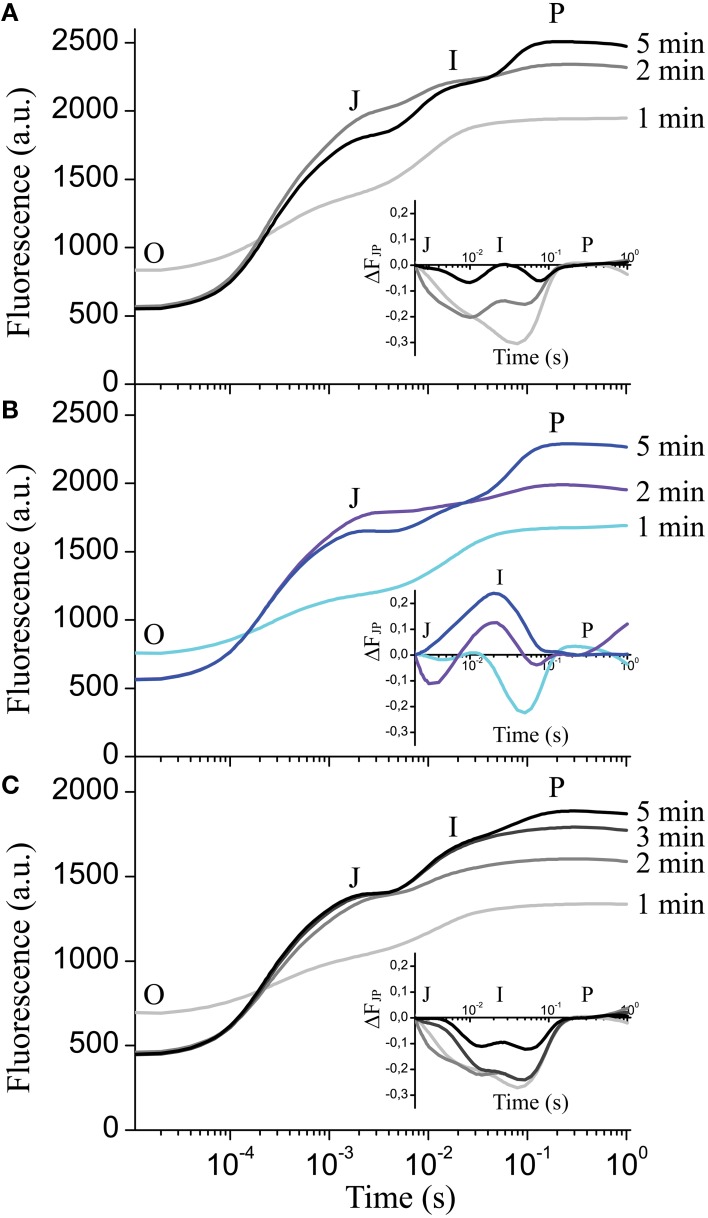
**Effect of dark adaptation interval on the fast chlorophyll ***a*** fluorescence ***OJIP*** transients**. *OJIP* transients were recorded on leaves of wild-type **(A)** and mutant plants **(B)**, following the indicated dark adaptation intervals. Insets show the curve difference (ΔF_JP_) of double normalized data at *J* (2 ms) and P, between 15 min dark adapted wildtype and 1, 2, and 5 min dark adapted wildtype **(A)** or *clce-2* **(B)**. **(C)** *OJIP* transients were recorded on wild-type leaves pre-incubated with 150 mM KCl in the light followed by the indicated dark adaptation intervals. Inset shows the curve difference (ΔF_JP_) of double normalized data at *J* and P between 15-min dark-adapted KCl-treated wildtype and 1, 2, 3, and 5-min dark-adapted KCl-treated wildtype. The plotted transients are means ± SEM (*n* = 5 plants).

Next we investigated if the formation of the *I* step in wildtype depends on the duration of dark adaptation interval preceding recording. Figure 6A shows the complete absence of the *I* peak after 1 min of dark adaptation with only the *J-P* peaks visible. After 2 min in darkness, the *I* step started to become visible, and after 5 min the complete *OJIP* kinetics could be observed (Figure 6A). The plotted ΔF_JP_ curve differences relative to 15-min dark adaptation (Figure 6A inset) indicate that 5 min in darkness is enough for complete formation of the *I* step in wildtype. The *clce-2* did not develop the *I* step after either 1 min or longer dark adaptation intervals (Figure 6B and inset). Pre-treatment with KCl delayed the appearance of the *I* step from 2 (Figure 6A) to 5 min in wildtype (Figure 6C), without reaching complete formation of the kinetics within the first 5 min (Figure 6C inset) relative to the 15 min dark-adapted leaves. These data suggest that formation of the *I* peak requires at least 5 min of dark adaptation in wildtype, possibly to allow for re-arrangement of electron transport components between PSII and PSI. KCl treatment appears to delay the formation of the *I* peak, since it possibly alters Cl^−^ distribution across thylakoids influencing electron transport.

**Figure 7:**
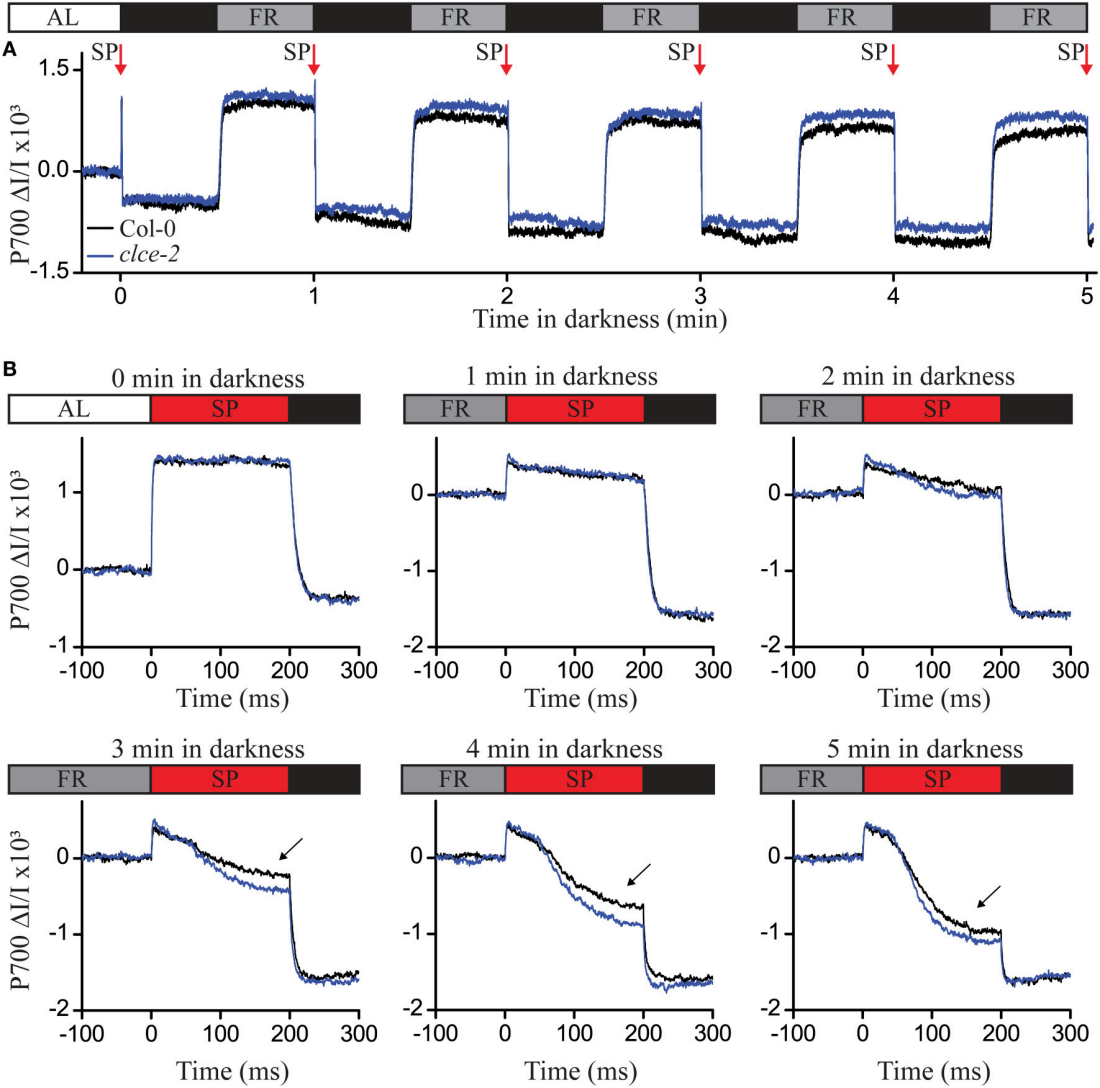
**P700 oxidation-reduction kinetics following different dark adaptation intervals**. Wild-type and mutant plants have been illuminated with actinic light (AL) of 120 μmol photons m^−2^ s^−1^ for 1 h before recording the P700 signal as difference in transmittance at 875 nm and 830 nm. The steady state P700 signal in the light was recorded for 15 s, followed by 5 cycles of 30 s (indicating the P700 reduced state) and 30 s of far-red (FR) illumination in darkness (indicating the P700 oxidized state) **(A)**. Before AL was switched off, and after each cycle of darkness and FR, a saturation pulse of 200 ms at 20,000 μmol photons m^−2^ s^−1^ (SP red arrow) was applied to record the P700 reduction kinetics **(B)**. The curves were normalized to the initial P700 values in light (**A**,**B**—0 min in darkness) or after the FR illumination **(B)**. Black arrows indicate differences in P700 reduction kinetics after 3, 4, and 5 min in darkness. The plotted data are means of measurements from 5 plants.

To test if PSI electron transfer was affected in the *clce-2* mutant, we used the same dark adaptation intervals as in Figure 6, and recorded P700 oxidation-reduction kinetics (Figure 7A). The reduction of P700^+^ was found more pronounced in the *clce* mutant as compared to wildtype after 3 min or longer period in darkness (Figure 7B). This suggests an accelerated electron transfer at PSI, which strengthens the possibility that this caused the lower fluorescence at the *I* step in Figure 6A. Taken together, the *OJIP* and P700 kinetics data indicate that in dark-adapted plants CLCe activity is important for Cl^−^ homeostasis of chloroplasts, which in turn influences optimal electron transport between PSII and PSI.

### State transition kinetics

**Figure 8:**
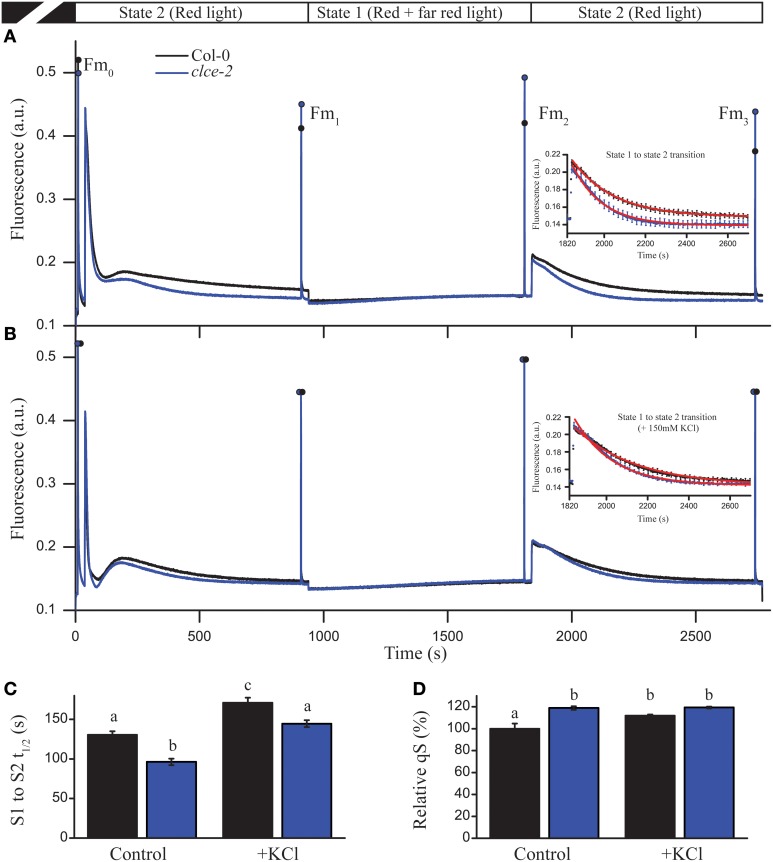
**State transition and light harvesting antenna changes in leaves**. State transition measurements were performed in untreated **(A)** and KCl-treated **(B)** wild-type and mutant leaves. Insets represent state 1 to state 2 transition kinetics. **(C)** The halftime (t_1∕2_) of fluorescence relaxation from S1 to S2 was calculated by fitting an exponential decay function on the fluorescence signal shown in insets. **(D)** Relative values of *qS* parameter indicate the ability of the chloroplast to adjust electron transfer to changes in light quality. The plotted data are means ±SEM (*n* = 4–5 plants). Different letters in panels **(C,D)** indicate statistically significant differences (ANOVA, *P* < 0.05).

We further investigated the consequences of AtCLCe loss-of-function on the plant ability to adapt to changes in light quality, and distribution of excitation energy between PSII and PSI (i.e., state transition) (Tikkanen et al., 2006). Steady-state Chl fluorescence levels of *clce-2* in “state 2 light” were found almost identical to the levels recorded in “state 1 light” (Figure 8A), whereas wild-type plants had a significant difference in fluorescence levels before the change of light from state 2 to state 1. These differences were no longer visible after treatment with KCl (Figure 8B). During illumination with “state 1 light,” steady-state fluorescence levels reached the same values in wildtype and *clce-2* mutant, however F_m_ (F_m2_) was significantly higher in *clce-2*, indicating a relatively larger PSII antenna size under state 1. Nevertheless, the state transition parameter *qT*, indicating the extent of changes in PSII antenna size, was not found significantly different between wildtype (0.116 ± 0.011) and *clce-2* (0.108 ± 0.003) or *clce-3* (0.114 ± 0.007). We additionally analyzed the kinetics for the transition from state 1 to state 2 (Figures 8A,B insets), which is known to result in decreased PSII antennae size (Bellafiore et al., 2005). The halftime (t_1∕2_) of PSII antenna detachment from PSII during S1 to S2 transition was found significantly lower in the mutant even after pre-treatment of the leaves with KCl (Figure 8C). Surprisingly, untreated wildtype leaves displayed the same t_1∕2_ as KCl-treated *clce-2*. Plant ability to adjust electron transfer to changes in light quality (*qS*) was found 20% higher in *clce-2* relative to wildtype in untreated leaves, whereas after treatment with KCl no significant difference was found between mutant and wildtype leaves, but remained 20% higher than untreated wildtype (Figure 8D).

### Circular dichroism analysis

**Figure 9:**
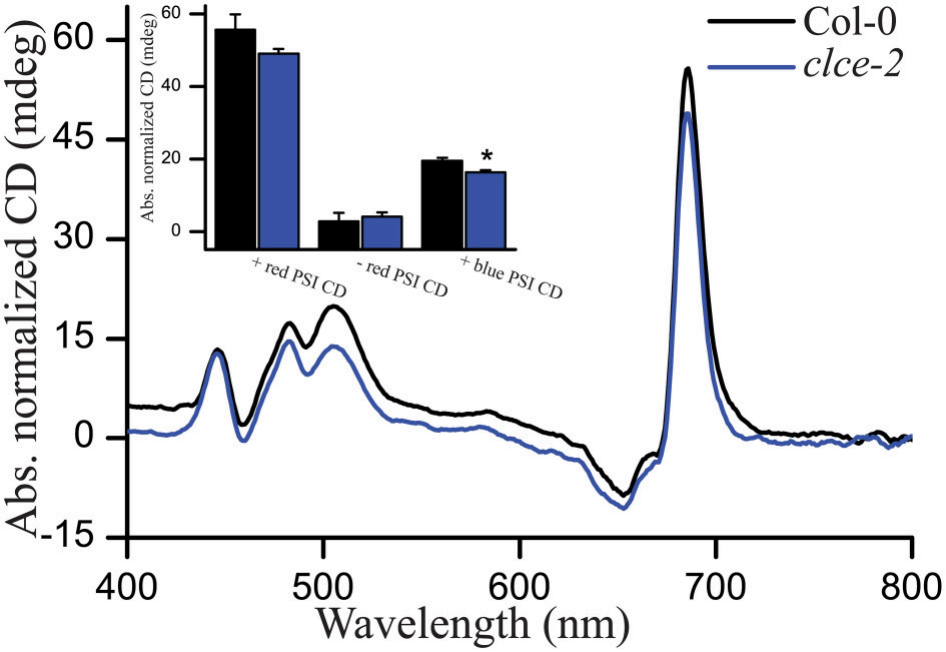
**Representative circular dichroism (CD) spectra of H_**2**_O-infiltrated detached leaves**. Insets are amplitudes of psi-type CD bands at around (+)505 nm (+blue), (−)675 nm (−red). and (+)690 nm (+red), with reference wavelengths at 550, 600. and 750 nm, respectively. Data are the means ± SEM (*n* = 3 plants). Asterisks indicate significant differences (ANOVA, *P* < 0.05).

We recorded CD spectra as a non-invasive method of studying the macro-organization of complexes in the thylakoid membrane (Garab and van Amerongen, 2009). In general, both wildtype and the *clce-2* mutant displayed typical CD spectra, suggesting no major differences in macro-organization of the complexes. Relatively lower values in the +red psi-type (PSI) and +blue PSI CD (Figure 9) were observed in the *clce-2* mutant as compared to wildtype, indicating a minor perturbation in the organization of PSII-LHCII macrodomains and/or a smaller domain-size compared to wildtype (Garab et al., 1991; Barzda et al., 1994).

### Chloroplast ultrastructure

**Figure 10:**
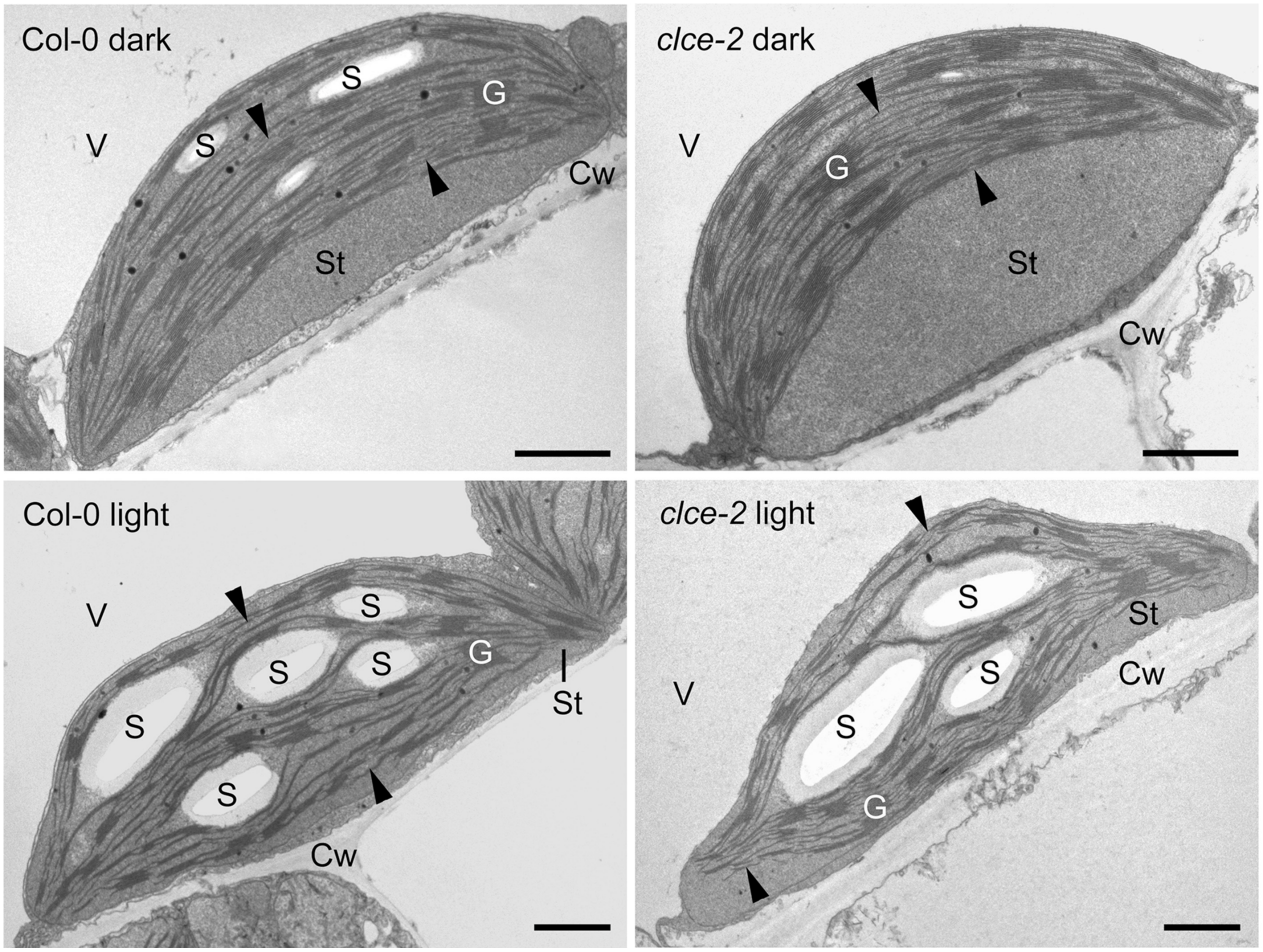
**Representative transmission electron micrographs of chloroplasts**. Leaves from 7-week-old wild-type and mutant plants that were dark-adapted for 16 h (“dark”) or further illuminated for 3 h at 120 μmol photons m^−2^ s^−1^ (“light”) were fixed in dim green light for electron microscopy. Arrowhead, stroma thylakoids; Cw, cell wall; G, granum; S, starch; St, chloroplast stroma; V, vacuole. Scale bar: 1 μm.

Representative TEM images (Figure 10) show that in leaves from the 16-h dark-adapted wild-type plants, chloroplasts were half-lens shaped with higher convexity than the elongated and flat chloroplasts observed in the 3-h light-adapted wild-type and *clce* plants. Dark-adapted *clce* plants had a more round shape and peculiar ultrastructural features: often a large thylakoid-free stromal zone was located next to the cell wall, and thylakoids with a bow-like arrangement were situated at the vacuolar side of the chloroplast (Figure 10). These features were prominent for ~75% of *clce* chloroplast sections, while they were also observed on ~50% of the wild-type chloroplast sections in the dark-adapted samples. However, in the latter the thylakoids had much less distorted, and less typical bow-like appearance, and the thylakoid-free stroma region was also smaller. The chloroplasts of 3-h light-adapted plants showed regular ultrastructure: their thylakoids were flat and the thylakoid network was arranged parallel to the cell wall, and only small thylakoid-free stroma regions were observed in ~30 and 10% of *clce* and wild-type chloroplast sections, respectively (Figure 10).

Detailed analyses of granum structure revealed no statistically significant differences between *clce* and wild-type plants either in the dark- or light-adapted states. The average granum diameters were around 450 and 400 nm in the dark- and in the light-adapted states, respectively. Similarly, the average number of appressed thylakoids per grana (i.e., granum height) was seven in both genotypes, and irrespectively from the light conditions.

There were no apparent differences in the starch contents of *clce* and wild-type chloroplasts in the light. However, assimilatory starch produced in the light almost completely disappeared during the dark-adaptation period in the *clce* mutant since only 50% of the analyzed chloroplast sections contained one small and very thin starch grain. Among the analyzed wild-type chloroplast sections from dark-adapted plants, 95% contained larger and often several starch grains. Taken together, the AtCLCe loss-of-function mutation influences the chloroplast ultrastructure in dark- but not in light-adapted plants.

## Discussion

### Role of AtCLCe in partial depolarization of the thylakoid membrane

Cl^−^ has been long considered to be the major counter-anion during electron transport-coupled H^+^ translocation, whose import into the lumen is expected to result in rapid partial depolarization of the thylakoid membrane (Hind et al., 1974). Cl^−^ is thought to be imported in the thylakoid lumen immediately after onset of illumination, and exported to the chloroplast stroma during transitions to dark (Hind et al., 1974). Malfunction of either the import or the export mechanism would result in altered Cl^−^ distribution within the chloroplast, hence altered ΔΨ during steady-state photosynthesis. As the only anion channel so far localized to thylakoids, AtCLCe has been hypothesized to be responsible for the partial depolarization of the thylakoid membrane in the light (Finazzi et al., 2015; Pottosin and Dobrovinskaya, 2015). Our data show small but significant increase in ΔΨ and total PMF in the *clce* mutants that occurred only at longer illumination time (≥5 min, Figures 2, 3A and Supplementary Figure 1). This supports the hypothesized role of AtCLCe in the partial depolarization of thylakoids, and in addition suggests that activation of AtCLCe may require factors dependent on the light exposure time. H^+^ conductivity through ATP synthase (${\text{g}}_{\text{H}}^{+}$) and proton flux ($\nu_{\text{H}}^{+}$) during illumination were also slightly increased (Figures 3B,C). Our data are in agreement with those of Kramer et al. (2003), who proposed that ATP synthase activity is driven by the amplitude of total PMF. Since the effects of the AtCLCe loss-of-function mutation on membrane depolarization and ATP synthase activity are only minor, additional, yet unidentified anion channels must contribute to the ion compensation of H^+^ uptake into the lumen during illumination.

Our data showing that AtCLCe can regulate ΔΨ suggest an electrogenic transport activity, i.e., the protein could work either as a channel or as an exchanger of anion and H^+^ at a stoichiometry of at least 2:1, which is the experimentally determined ratio for the algal CLC (Feng et al., 2010). If AtCLCe worked as an anion/H^+^ exchanger, then it was expected to play an active role in mediating pH homeostasis or to act as a H^+^ leak to reduce lumenal pH. The mechanism of transport is unknown as well as whether there is coordination between the ATP synthase and AtCLCe to generate the required transmembrane pH gradient. Such coordination might be indirect via the same protein regulator (e.g., Batelli et al., 2007).

### Role of AtCLCe in regulation of PSII efficiency and photoprotection

Cl^−^ ions are thought to be important for channeling H^+^ from the oxygen-evolving complex of PSII to the thylakoid lumen (Guskov et al., 2009; Umena et al., 2011). Additionally, *in vitro* experiments in media depleted of Cl^−^ showed that PSII particles harbor unstable oxygen-evolving complex (Nash et al., 1985). Under our experimental conditions, PSII efficiency of the *clce* mutants in the light [Φ (*II*)] was found unchanged, and only during the subsequent dark recovery phase was lower relative to wildtype (Figures 4C,D). These observations suggest that AtCLCe loss-of-function mutation affects PSII activity during dark adaptation rather than during illumination. The cause behind the observed changes in PSII activity could be the thylakoid ultrastructural changes in dark-adapted plants discussed below. Moreover, the lower *F*_*v*_*/F*_*m*_ and *PI* parameters in the *clce* mutants (Table 1), further indicate an unfavorable organization of the electron transport components in darkness, since these parameters can only be determined correctly after subsequent dark adaptation. The higher steady-state *NPQ* in *clce* (Figures 4A,B) cannot be easily explained by the lower ΔpH contribution to PMF partitioning (Figure 2), however, other unknown factors related to the ΔΨ component may play a role.

### Role of AtCLCe in regulation of electron transport via re-arrangement of thylakoid network

Fast chlorophyll fluorescence (*OJIP*) kinetics is a useful tool to obtain information about functioning of the electron transport chain. The observed decrease in fluorescence levels at the *I* step seen in the *OJIP* kinetics of *clce* mutants (Figure 5A, Table 1) together with the more pronounced P700^+^ reduction (Figure 7) indicate accelerated electron transfer at PSI (Schansker et al., 2005). The extended time to reach *F*_*m*_ suggests that it takes twice as long in the mutants as compared to wildtype to completely close all PSII centers (Figure 5A inset). Moreover, the reduced variable fluorescence at the *J* (*V*_*J*_) and *I* (*V*_*I*_) steps, as well as the double number of Q_A_ reduction events (*N*) (Table 1) in the *clce* mutants further suggest an accelerated electron transfer between PSII and PSI as compared to wildtype.

The chloroplast thylakoid lumen undergoes swelling during illumination and shrinkage in darkness (Kirchhoff et al., 2011; Yoshioka-Nishimura et al., 2014). This is thought to be driven mainly by Cl^−^ influx into the thylakoid lumen in the light and efflux in darkness (Kirchhoff, 2013). Expansion of the lumen is necessary for efficient electron transport between Cyt b_6_*f* and PSI via the soluble protein plastocyanin (PC), whose diffusion was proposed to depend on physical space. Additionally, lumen swelling is important for PSII repair (Kirchhoff et al., 2011) and state transition (Chuartzman et al., 2008). Based on this knowledge and our data, we hypothesized that the *clce* mutants may retain partially swollen thylakoids even after dark adaptation. A swollen lumen would allow for increased mobility of PC, facilitating electron transport from Cyt b_6_*f* to PSI (Kirchhoff et al., 2011). However, no differences in shrinkage/swelling of the thylakoid lumen or significant alterations in granum height and diameter could be resolved by TEM between *clce* and wild-type plants (Figure 10). The grana and stroma thylakoid network of wildtype and mutant chloroplasts was flat and arranged parallel to the cell walls to maximize photosynthetic energy capture under relatively low light conditions (120 μmol photons m^−2^ s^−1^). Nevertheless, dark-adapted *clce* chloroplasts had a more pronounced, special bow-like arrangement of the thylakoid network. Similar arrangement of the thylakoid system was reported for the chloroplasts of other photosynthetic species under different stress conditions, including excess of heavy metals that cause disturbances in the ion homeostasis of chloroplasts (e.g., reviewed by Solymosi and Bertrand, 2012). We suggest that the peculiar chloroplast shape and thylakoid arrangement in the *clce* mutant in darkness could be a mechanism to overcome altered Cl^−^ homeostasis within the chloroplast. The disturbances in chloroplast ultrastructure may in turn alter the functioning of the photosynthetic electron-transport chain. The *clce* mutant can adapt to light similarly to wildtype as indicated by similar thylakoid ultrastructural changes. This suggests that additional Cl^−^ transport mechanism must exist in thylakoids in the light. This unknown mechanism may be also involved in the restoration of wildtype *OJIP* kinetics by KCl pre-treatment (Figure 5B). Similar treatment with KNO_3_ did not restore the wild-type kinetics (Figure 5C), suggesting that either ${\text{NO}}_{3}^{-}$ homeostasis was not altered in the *clce* mutant or that changes in ${\text{NO}}_{3}^{-}$ homeostasis did not affect photosynthesis in the *clce* mutants.

The altered *OJIP* kinetics observed in *clce* leaves could also be reproduced in wildtype following dark adaptation for 1 min (Figures 6A,B). More specifically, this short dark adaptation resulted in a complete absence of the *I* step in both wildtype and mutant, and only the *OJP* kinetics were visible. In leaves treated with KCl, we observed a slower formation of the *I* step, since it became visible only after 5 min (Figure 6C and inset), and even after 15 min the amplitude was still lower relative to untreated leaves (Figures 5A,B). The observation of a delay in formation of the *I* step upon KCl treatment suggests that ion homeostasis in darkness is important for a proper re-arrangement of the electron transport chain components. We also found a faster transition of *clce-2* from state 1 to state 2 (Figures 8A–C) as well as the higher *qS* values (Figure 8D). A possible explanation is that Cl^−^ ions may directly influence the electrostatic interactions of LHCII with PSII or PSI, which in turn affect their ability to migrate, also leading to an unfavorable arrangement in darkness. The macro-organization of complexes and the structural stability of the chiral macro-domains have been shown to depend on the ionic strength of the medium (Garab et al., 1991; Cseh et al., 2000). Alternatively, a long-lived membrane potential in darkness due to sustained Cl^−^ ions trapped in the thylakoid lumen may also have a negative effect on the dark relaxation of the electron transport chain.

To conclude, our findings suggest that AtCLCe functions in Cl^−^ homeostasis within the chloroplast leading to re-arrangement of the electron transport chain in thylakoids after transition from light to dark. Changed Cl^−^ distribution across thylakoids may be one of the strategies to ensure maximum quantum yields and balance photochemical utilization with photoprotection by *NPQ* upon light-to-dark and dark-to-light transitions. We propose a minor role for AtCLCe in light-driven Cl^−^ import into the thylakoid lumen, and a major role in Cl^−^ export to the chloroplast stroma upon dark adaptation. The major Cl^−^ import mechanism driven by membrane potential produced during illumination remains to be identified. Cl^−^ export by AtCLCe in darkness would hence be driven by the inversed membrane potential across the thylakoid membrane, which occurs when light is switched off, and would facilitate re-arrangements of the electron transport chain components in thylakoids.

### AtCLCe—a Cl^−^ channel or an ${\text{NO}}_{3}^{-}$/H^+^ exchanger?

AtClCe has in general a modest homology with the other plant CLCs, and forms a distinct family branch together with AtCLCf (Barbier-Brygoo et al., 2011). Phylogenetic analyses indicated the presence of homologs in green algae, brown algae, diatoms and cyanobacteria, sharing 25–35% identity with AtCLCe, but thus far none have been characterized (Pfeil et al., 2014).

CLCs were initially thought to be involved in Cl^−^ transport after the first CLC was cloned, which was the voltage-dependent Cl^−^ channel of torpedo fish (CLC-0; Jentsch et al., 1990). Crystal structures revealed bacterial CLCs as secondary active transporters that exchange Cl^−^ and H^+^ with a 2:1 stoichiometry (Accardi and Miller, 2004). Animal CLCs were found to function as Cl^−^ channels at the plasma membrane or as 2Cl^−^/1H^+^ exchangers in organellar membranes (Jentsch, 2015). Even though they catalyze distinct transport reactions, they share the basic protein architecture. More specifically, CLC proteins are homodimers with separate ion pathways within each monomer (Dutzler et al., 2002). Each monomer consists of a transmembrane component, which forms the ion transportation pathway, and in the case of eukaryotic members, also of a cytosolic cystathionine beta-synthase (CBS) domain component, which binds nucleotides and regulates the transmembrane component.

Multiple sequence alignment of AtCLCe with three CLCs from prokaryotes and eukaryotes indicated 17.9% identity with AtCLCa (De Angeli et al., 2006), 20.2% with CLC from *Cyanidioschyzon merolae* (Feng et al., 2010), and 23.3% with the CLCa from *Escherichia coli* (Dutzler et al., 2002; Supplementary Figure 2). AtCLCe sequence lacks many residues in the anion selectivity filter conserved in most CLCs (Dutzler et al., 2002; Feng et al., 2010). It does not harbor either the conserved serine in the Cl^−^ binding site of crystallized CLCs or the proline residue, shown to be crucial for preference of ${\text{NO}}_{3}^{-}$ vs. Cl^−^ in AtCLCa (Wege et al., 2010). Nevertheless, the positively charged residue (lysine) at this position may still be able to coordinate Cl^−^ in the transportation pathway, but reduces the chances of a preference for transport of ${\text{NO}}_{3}^{-}$. Thus the previous observation that *clce* mutants displayed altered nitrate accumulation (Monachello et al., 2009) could be an indirect effect due to possible alteration in the expression of ${\text{NO}}_{3}^{-}$/H^+^ exchangers from the AtCLC family. In addition, our data showing similar *OJIP* phenotype in untreated and KNO_3_-treated *clce* mutants (Figure 5) indicates that even if AtCLCe would play a role in nitrate homeostasis, this did not affect photosynthetic electron transport in thylakoids. Instead, AtCLCe appears to function in Cl^−^ homeostasis, which affects electron transport via thylakoid re-arrangements.

AtCLCe sequence contains the so-called “gating glutamate” (Supplementary Figure 2), which is conserved in almost all CLCs and which protonation opens the Cl^−^ transportation pathway (Feng et al., 2010). However, AtCLCe sequence does not contain the “proton glutamate” important for H^+^ translocation, which could indicate that this CLC member is not an anion H^+^ exchanger. Instead, at the “proton glutamate” position, a serine residue is present in AtCLCe and a threonine in algal CLC, which was shown to still function as an exchanger (Feng et al., 2010). In addition to serine and the gating glutamate, a tyrosine residue is involved in coordination of the Cl^−^ ion in the translocation pathway. This residue is replaced by a threonine in AtCLCe, which is also polar and harbors a hydroxyl group important for coordination. Finally, the aspartate residue involved in ATP binding in the CBS domain (De Angeli et al., 2009) is replaced by a cysteine in AtCLCe. As reviewed by Pottosin and Dobrovinskaya (2015), electrophysiological studies of AtCLCe are required to determine the Cl^−^ vs. ${\text{NO}}_{3}^{-}$ selectivity and channel vs. exchanger mechanism of transport for this protein. This will also allow to establish whether AtCLCe is responsible for the channel activity previously reported in thylakoids (Schönknecht et al., 1988).

## Author contributions

AH, BL, and CS conceived the study and designed the experiments. AH carried out chlorophyll fluorescence, P700, ST, and ECS measurements. HN conducted screening of the mutants and RT-PCR. OZ carried out CD analyses. KS carried out TEM analyses. AH and CS wrote the manuscript. All authors helped to edit the manuscript.

### Conflict of interest statement

The authors declare that the research was conducted in the absence of any commercial or financial relationships that could be construed as a potential conflict of interest.

## Abbreviations

CD: circular dichroism
Chl: chlorophyll
CLC: chloride channel
Cyt: cytochrome
F_v_/F_m_: maximum quantum yield of photosystem II photochemistry
FR: far red
LHC: light harvesting complex
ΔΨ: membrane potential
NPQ: non-photochemical quenching
P700: primary donor of photosystem I
PC: plastocyanin
PI: performance index
Δ pH: pH gradient
g_H_^+^: proton conductivity through ATP synthase
ν_H_^+^: proton flux
PMF: proton motive force
PS: photosystem
Φ(II): PSII efficiency
ST: state transition
TEM: transmission electron microscopy.
