# Supplementary material for: The Arabidopsis Thylakoid Chloride Channel AtCLCe Functions in Chloride Homeostasis and Regulation of Photosynthetic Electron Transport
Source: Front Plant Sci. 2016 Feb 9;7:115. doi: 10.3389/fpls.2016.00115 (PMC4746265; doi:10.3389/fpls.2016.00115)
Supplement: Supplementary file 1 [file Presentation1.pdf]

Supplementary Material

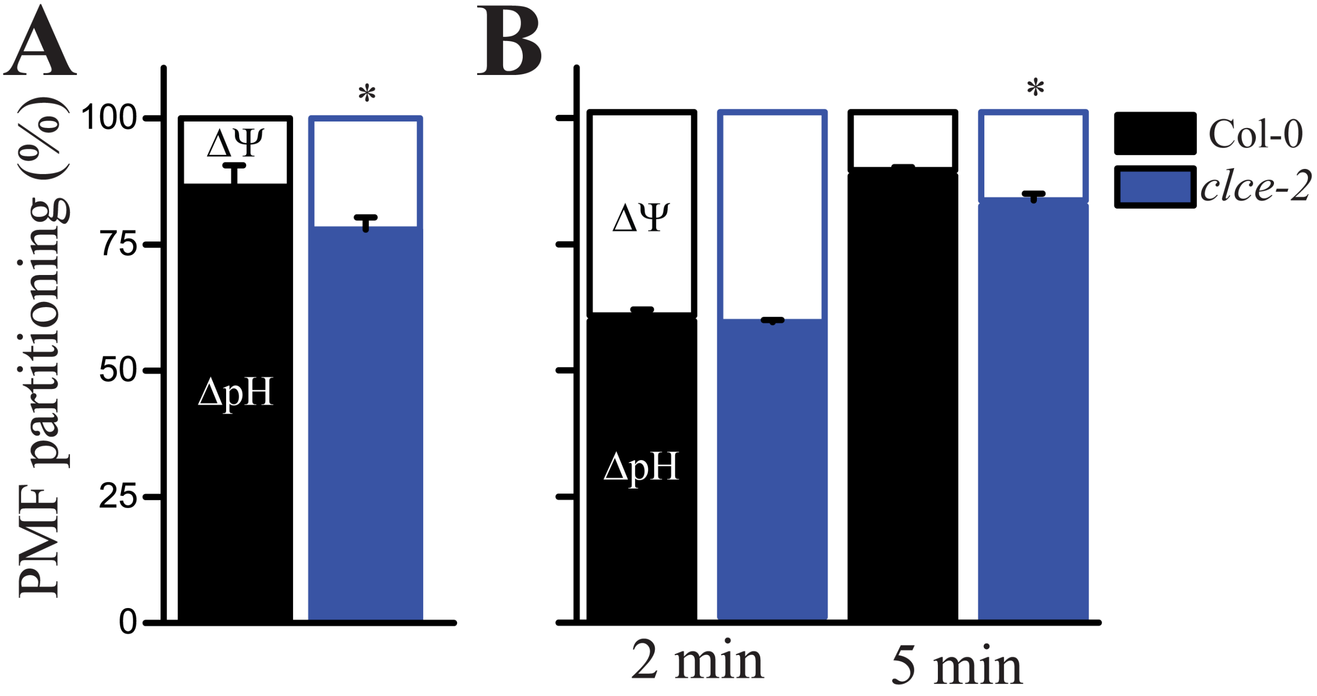

Supplementary Figure 1. Composition of proton motive force (PMF).

(**A**) PMF partitioning into membrane pH gradient (ΔpH) and membrane potential (ΔΨ) after 10 min illumination at 100 μmol photons m^{-2} s^{-1}.

(**B**) PMF partitioning after 2 or 5 min illumination at 650 μmol photons m^{-2} s^{-1}. The plotted data are means ± SEM (n=5-6 plants). Asterisks indicate statistical differences according to ANOVA ($P<0.05$).

```
CLC_cyame       ---------MSSKITGTLEVDYGLLDSMGNLARHPTPVGTR------ASESSQQISLEET
CLCa_arath      MDEDGNLQISNSNYNGEEEGEDPENNTLN------QPLLKR-----HRTLSSTPLALV--
CLCe_arath      -----------------MAATLPLCAALR------SPVSSRRFAPIHKTDVPFQFNVVLS
CLCa_ec         -----------------MKTDTPSLE---------TPQAAR-------------------
                                                    *   *

CLC_cyame       ESGLRLAPYDTGFDANERSYGSVSPRDAYRCWNALGSCTPRGSLMYLLRLVCFL-TLLGV
CLCa_arath      --------GAKVSHIESLDYEINENDLFKHDWRSRSKAQVFQYIFLKWTLACLVGLFTGL
CLCe_arath      PFFGSVAIGGRIFPRLPAAKQETDQDEVGFDQQPSQE--------LAIASACLVGVLTGV
CLCa_ec         -----------------LRRRQLIRQLLERDKTPLA----------ILFMAAVVGTLVGL
                               . .                ...:  : *:

CLC_cyame       TAALFIFAV------------DLAVHGLEELRM-KISRLAGRFAGYILY-VVSGVALCLL
CLCa_arath      IATLINLAV-------------ENIAGYKLLAVGYYIAQDRFWTGLMVF-TGANLGLTLV
CLCe_arath      SVVLFNNCVHLLRDFSWDGIPDRGASWLREAPIGS------NWLRVILVPTIGGLVVSIL
CLCa_ec         AAVAF----------------DKGVAWLQNQRMGALVHTADNYPLLLTVAFLCSAVLAMF
                 .. :                           :         :   :      .  : :.

                      anion selectivity                        gating Glu
CLC_cyame       STFWCAVLSTEAEGSGLPQMKSILSGFYDKMRSALELRVLFAKALGLICAIGGGLPVGWE
CLCa_arath      ATVLVVYFAPTAAGPGIPEIKAYLNGI--DTPNMFGFTTMMVKIVGSIGAVAAGLDLGKE
CLCe_arath      NQL------RESAGKSTGDSHSSLDRVKAVLRP-------FLKTVAACVTLGTGNSLGPE
CLCa_ec         GYFLVRKYAPEAGGSGIPEIEGALE----DQRPVRWWRVLPVKFFGGLGTLGGGMVLGRE
                  .        : * .  :  . *.                 * ..   ::. *  :* *

CLC_cyame       GPNVHIACII-------AHQFYRLG--VFKELCTDRALRLQTLAAACAVGLASSFGAPLG
CLCa_arath      GPLVHIGSCIASLLGQGGPDNHRIKWRWLRYFNNDRD-RRDLITCGSASGVCAAFRSPVG
CLCe_arath      GPSVEIGASI----AKGVNSLFN-K--------SPQT-GFSLLAGSAAGISSGFNAAVA
CLCa_ec         GPTVQIGGNI----GRMVLDIFRLK--------GDEA-RHTLLATGAAAGLAAAFNAPLA
                ** * *.  *         . ..                   :: ..* *:.:.* :.:.

                   proton Glu
CLC_cyame       GVLYSIETIASFYLVQAFWKGVLSALSGAIVYELLYT---------TPLVEAFEGTNFDA
CLCa_arath      GVLFALEEVATWWRSALLW---RTFFSTAVVVVVLRAFIEICNSGKCGLFGSGGLIMFDV
CLCe_arath      GCFFAVESV--------LWPSSSTDSSTSLPNTTSMVILSAVTASVVSEIGLGSEPAFKV
CLCa_ec         GILFIIEEMRPQFRYTLIS--IKAVFIGVIMSTIMYRIF----NHEVALIDVG-----KL
                * :: :* :        :     :     :                   .        .

CLC_cyame       SDV----SRTQTLLYAILGALMGVLGALFIRCVRSI---YELRMRHYPGTNRYF-LVGVV
CLCa_arath      SHVEVRYHAADIIPVTLIGVFGGILGSLYNHLLHKVLRLYNLINQK-GKIHKVL-LSLGV
CLCe_arath      PDYDFR-SPGELPLYLLLGALCGLVSLALSRCTSSMTSAVDSLNKDAGIPKAVFPVMGGL
CLCa_ec         SDA----PLNTLWLYLILGIIFGIFGPIFNKWVLGM---QDLLHRVHGGNITKW-VLMGG
                .               ::* : *:..    .    :    :   .          :

CLC_cyame       ALFASALQYPFRLFA-----------LDPRATINDLFKAVPL------------------
CLCa_arath      SLFTSVCLFGLPFLAECKPCDPSIDEICPTNGRSGNFKQFNCPNGYYNDLSTLLLTTNDD
CLCe_arath      SVGIIALVYPEVLYW--------------------GFQNVDI------------------
CLCa_ec         AIGGLCGLLGF---------------VAP-ATSGGGFNLIPI------------------
                ::                                  *: .

CLC_cyame       --------YQTDHFGWTELILMPIIKFILVALSIGLPLPAGVFVPSFLIGAGFGRLYGEL
CLCa_arath      AVRNIFSSNTPNEFGMVSLWIFFGLYCILGLITFGIATPSGLFLPIILMGS----AYGRM
CLCe_arath      ---LLEKRPFVKGLSADLLLQLVAVKIAATAWCRASGLVGGYYAPSLFIGGAAGMAYGKF
CLCa_ec         --------ATAGNFSMGMLVFIFVARVITTLLCFSSGAPGGIFAPMLALGTVLGTAFGMV
                             :.   *  :            .    .* : * : :*      :* .

CLC_cyame       MRVVFGNAI----------VPGSYAVVGAAAFTAGVTRA-LSCAVIIFEVTGQIRHLVPV
CLCa_arath      LGTAMGSYTNI--------DQGLYAVLGAASLMAGSMRMTVSLCVIFLELTNNL-LLLPI
CLCe_arath      IGLALAQNPDFNLSILEVASPQAYGLVGMAATLAGVCQVPLTAVLLLFELTQDYRIVLPL
CLCa_ec         AVELFPQYHL---------EAGTFAIAGMGALLAASIRAPLTGIILVLEMTDNYQLILPM
                    : .                :.: * .:  *.  .  ::  ::.:*:* :   ::*:

                      anion selectivity
CLC_cyame       LISVLLAV-IVGNAFNRSLYETLVLMKHLPYMPILRRDRSPEMTAREIMHPIEGEPHLFP
CLCa_arath      TMFVLLIAKTVGDSFNLSIYEIILHLKGLPFLE---ANPEPWMRNLTVGELNDAKPPVVT
CLCe_arath      LGAVGMSSWITSGQSKRQETRETKETRKRKSQEAV-QSLTSSDDESSTNNLCEVESSLCL
CLCa_ec         IITGLGATLLAQFTGGKPLYSAIL------------------------------------
                          .

CLC_cyame       DSEPQHIKGILE--KFPN--RLVFPVIDANGYLLGAISRKEIVDRLQHVLEDVPEPIAGH
CLCa_arath      LNGVEKVANIVD--VLRNTTHNAFPVLDGADQNTGTELHGLILRAHLVKVL---------
CLCe_arath      DDSLNQSEELPKSIFVSEAMRTRFATVMMSTSLEEALTRMLIEKQSCALIVD--------
CLCa_ec         ------------------------------------------------------------

CLC_cyame       RTLVLLDAADLSENIEGLVDETPSGEHSSKGKRTATVLEPTS----------SLVVPCDV
CLCa_arath      KKRWFLNEKRRTEEWEVREKFTPVELAEREDNFDDVAITSSEMQLYVDLHPLTNTTPYTV
CLCe_arath      PDNIFLGILTLSDIQEFSKARKEGNNRPKDIFVNDICSRSGG----------KCKVPWTV
CLCa_ec         -------ARTLAKQEAEQLARSKAASASENT-----------------------------
                           :.        .       .

                                                       ATP binding site
CLC_cyame       SPI--VVTSYSLVRQLHFLFVMLMPSMIYVTERGKLVGIVEREDVAYGYREPEQTS----
CLCa_arath      VQSMSVAKALVLFRSVGLRHLLVVPKIQ-ASGMSPVIGILTRQDLR-AYNILQAFPHLDK
CLCe_arath      TPDMDLLAAQTIMNKHELSHVAVVSGSI-DAPRIHPVGVLDRECITLTRRALATRMYLLN
CLCa_ec         ------------------------------------------------------------

CLC_cyame       -------
CLCa_arath      HKSGKAR
CLCe_arath      SLYL---
CLCa_ec         -------
```

Supplementary Figure 2. Multiple sequence alignment of CLCs using MUSCLE 3.8.

The aligned sequences are for CLC from *Cyanidioschyzon merolae* (CLC_cyame), CLCa from *Arabidopsis thaliana* (CLCa_arath), CLCe from *Arabidopsis thaliana* (CLCe_arath), and CLCa from *Escherichia coli* (CLCa_ec). The sequence of CLCe displayed 20.2% identity with CLC_cyame, 17.9% with CLCa_arath, and 26.3% with CLCa_ec. The putative chloroplast-transit peptide is marked in green. The positions of important residues for anion transport in characterized CLCs are framed. Where conserved, those residues are coloured and the name of the proposed function is given.
